# Role of the cytoplasmic isoform of RBFOX1/A2BP1 in establishing the architecture of the developing cerebral cortex

**DOI:** 10.1186/s13229-015-0049-5

**Published:** 2015-10-20

**Authors:** Nanako Hamada, Hidenori Ito, Ikuko Iwamoto, Rika Morishita, Hidenori Tabata, Koh-ichi Nagata

**Affiliations:** Department of Molecular Neurobiology, Institute for Developmental Research, Aichi Human Service Center, 713-8 Kamiya, Kasugai Aichi, 480-0392 Japan

**Keywords:** Rbfox1, Neuronal migration, Axon growth, Synapse formation, Corticogenesis

## Abstract

**Background:**

RBFOX1 (also known as FOX1 or A2BP1) regulates alternative splicing of a variety of transcripts crucial for neuronal functions. Physiological significance of RBFOX1 during brain development is seemingly essential since abnormalities in the gene cause autism spectrum disorder (ASD) and other neurodevelopmental and neuropsychiatric disorders such as intellectual disability, epilepsy, attention deficit hyperactivity disorder, and schizophrenia. *RBFOX1* was also shown to serve as a “hub” in ASD gene transcriptome network. However, the pathophysiological significance of *RBFOX1* gene abnormalities remains to be clarified.

**Methods:**

To elucidate the pathophysiological relevance of Rbfox1, we performed a battery of in vivo and in vitro analyses of the brain-specific cytoplasmic isoform, Rbfox1-iso2, during mouse corticogenesis. In vivo analyses were based on in utero electroporation, and the role of Rbfox1-iso2 in cortical neuron migration, neurogenesis, and morphology was investigated by morphological methods including confocal laser microscope-assisted time-lapse imaging. In vitro analyses were carried out to examine the morphology of primary cultured mouse hippocampal neurons.

**Results:**

Silencing of Rbfox1-iso2 in utero caused defects in the radial migration and terminal translocation of cortical neurons during corticogenesis. Time-lapse imaging revealed that radial migration was apparently impaired by dysregulated nucleokinesis. Rbfox1-iso2 also regulated neuronal network formation in vivo since axon extension to the opposite hemisphere and dendritic arborization were hampered by the knockdown. In in vitro analyses, spine density and mature spine number were reduced in Rbfox1-iso2-deficient hippocampal neurons.

**Conclusions:**

Impaired Rbfox1-iso2 function was found to cause abnormal corticogenesis during brain development. The abnormal process may underlie the basic pathophysiology of ASD and other neurodevelopmental disorders and may contribute to the emergence of the clinical symptoms of the patients with *RBFOX1* gene abnormalities.

**Electronic supplementary material:**

The online version of this article (doi:10.1186/s13229-015-0049-5) contains supplementary material, which is available to authorized users.

## Background

RNA-binding Fox1 (Rbfox1), also known as Fox-1 or ataxin-2-binding protein 1 (A2BP1), was first identified as a binding partner for ataxin-2 [1]. Rbfox1 is dominantly expressed in the neuronal tissues, muscle, and heart and has been reported to play a pivotal role in alternative splicing of genes critical for neuronal development such as calcitonin/calcitonin gene-related peptide, CaV1.2 voltage-gated calcium channels, and *N*-methyl-d-aspartate (NMDA) receptor 1 [2–5].

Accumulating clinical reports strongly suggest the involvement of *RBFOX1* in various neurodevelopmental disorders. Fluorescence in situ hybridization (FISH), array comparative genomic hybridization (aCGH), and genome-wide linkage studies (GWAS) have been demonstrated that *RBFOX1* is associated with autism spectrum disorder (ASD) and other neuropsychiatric disorders including intellectual disabilities (IDs), epilepsy, attention deficit hyperactivity disorder (ADHD), and schizophrenia [6–15]. In addition, RBFOX1 target transcripts predicted by bioinformatics are significantly overlapping with genes implicated in ASD [16]. Furthermore, *RBFOX1* was found to serve as a “hub” in gene transcriptome networks based on the weighed gene coexpression network analysis and microarray profiling of postmortem ASD patient brains [17]. Interestingly, abnormal reduction of RBFOX1 expression in a subset of ASD patient brains was correlated with altered splicing of its predicted targets including two ASD-related genes (*GRIN1* and *MEF2C*), although the pathophysiological relevance of the splicing variation remains to be elucidated [17]. In addition, massive splicing changes were detected in 48 genes in a group of ASD patient brains where downregulation of RBFOX1 was estimated [18].

In this report, we focused on an Rbfox-1 isoform, Rbfox1-iso2, which is distributed dominantly in the cytoplasm of cortical neurons [19, 20], and analyzed its function in brain development. While Rbfox1-iso2 appeared not to be involved in neuronal cell proliferation, its deficiency caused defects in migration, axon growth, and dendrite development of excitatory neurons during corticogenesis. The results obtained here indicate that Rbfox1-iso2 may play a crucial role in cerebral cortex development independently of the nuclear isoform Rbfox1-iso1, and functional defects of Rbfox1-iso2 may be related to the etiology of ASD and neurodevelopmental disorders with the gene abnormalities.

## Methods

### Study approval

We followed the Fundamental Guidelines for Proper Conduct of Animal Experiments and Related Activity in Academic Research Institution under the jurisdiction of the Ministry of Education, Culture, Sports, Science and Technology, and all of the protocols for animal handling and treatment were reviewed and approved by the Animal Care and Use Committee of Institute for Developmental Research, Aichi Human Service Center (approval number, M10).

### Plasmid construction

Mouse (m)Rbfox1-iso2(A2BP1-A030) and Rbfox1-iso1(A2BP1-A016) cDNAs in pCS-MT vector with Myc-tag were supplied by Dr. S. Kawamoto (NIH, MD)[19, 21] and constructed into pCAG-Myc vector (Addgene Inc., Cambridge, MA). pCAG-PACKmKO1 and pβAct-EGFP were gifts from Drs. F. Matsuzaki (RIKEN, Kobe, Japan) [22] and S. Okabe (Univ. Tokyo, Japan) [23], respectively. pCAG-histone 2B (H2B)-EGFP was used to label chromosomes. pCAG-M-Cre was from Dr S. Miyagawa (Univ. Osaka, Japan) [24], and pCALNL (loxP-neomycin-loxP)-GFP was made from pCALNL-DsRed (Addgene Inc.). The following target sequences were inserted into pSuper-puro RNAi vector (OligoEngine, Seattle, WA): mRbfox1-iso1, GACTAGGAGCCATGCTGAT (1098-1106 in mRbfox1-iso1) [20]; mRbfox1-iso2, GATGAAATTTCTTGTAACA (1018-1036 in mRbfox1-iso2); mRbfox1-iso1/2, GTAAAATCGAGGTTAATAA (548-566 in mRbfox1-iso1). Numbers indicate the positions from translational start sites. We named these vectors as pSuper-mRbfox1-iso1, pSuper-mRbfox1-iso2, and pSuper-mRbfox1-iso1/2. For the control RNAi experiments, we used pSuper-H1.shLuc, a shRNA designed against luciferase (CGTACGCGGAATACTTCGA) [25] as well as pSuper vector, both of which had no effects on the phenotypes analyzed here. To make an RNAi-resistant version of mRbfox1-iso2, mRbfox1-iso2R, silent mutations were introduced in the target sequence as underlined (GACGAGATCAGCTGCAATA in mRbfox1-iso2).

### Antibodies

Polyclonal anti-A2BP1(Rbfox1) and anti-Sept11 were generated as described [20, 26]. Polyclonal rabbit antibodies for green fluorescent protein (GFP) and red fluorescent protein (RFP) were from MBL (Nagoya, Japan) and Rockland Immunochemicals (Gilbertsville, PA), respectively. Anti-GFP (Chicken) was purchased from AVES Labs (Tigard, OR). Rabbit polyclonal anti-Ki67 was from Thermo Scientific Japan (Yokohama, Japan).

### Cell culture, transfection, western blotting, and immunofluorescence

COS7 and mouse primary neurons were cultured essentially as described [27, 28]. Transfection was done with Lipofectamine 2000 (Life Technologies Japan, Tokyo). Western blotting was carried out as described [20]. We used Sept11, a ubiquitously expressed cytoskeletal protein [26], as a loading control. Immunofluorescence analyses were performed with Alexa Fluor 488- or 568-labeled IgG (Life Technologies Japan) as a secondary antibody [29]. Fluorescent images were obtained using FV-1000 confocal laser microscope (Olympus, Tokyo, Japan).

### In utero electroporation and time-lapse imaging

Pregnant ICR mice were purchased from SLC Japan (Shizuoka, Japan). In utero electroporation was performed essentially as described [30]. Each RNAi vector (1 μg) was electroporated with GFP vector (0.5 μg) in a total volume of 1 μl. Live-imaging analyses were performed essentially as described [31].

### Quantitative analysis of neuronal migration

The coronal sections of cerebral cortices containing the labeled cells were classified into five bins and the intermediate zone (IZ) as described previously [32].

### EdU incorporation experiments

pCAG-H2B-EGFP vector was electroporated in utero into embryos with pSuper vector (control) or pSuper-mRbfox1-iso2 at embryonic day (E)14. Forty hours after electroporation, pregnant mice were given an intraperitoneal injection of 5-ethynyl-2′-deoxyuridine (EdU) at 25 mg/kg body weight. One hour after injection, brains were fixed with 4 % paraformaldehyde and frozen sections were made. GFP and EdU were detected with anti-GFP and Alexa Fluor555 azide (Life Technologies Japan), respectively. When the ratio of EdU/Ki67/GFP-triple-positive cells to EdU/GFP-double-positive ones was determined, EdU injection was done at 24 h after electroporation. Brains were then sectioned after 24 h and subjected to detection for EdU, Ki67, and GFP.

### Quantitative analysis of axon growth

RFP signal intensity of the callosal axons was measured in a 170 × 200 μm rectangle on both the ipsilateral (green) and contralateral (white) sides at the positions indicated in Fig. 5a (*left* panel). The ratio of the axonal RFP signals in the contralateral side to the corresponding ipsilateral side was calculated.

### Quantitative analysis of spine morphologies

Transfected neurons were immunostained with anti-GFP and chosen randomly. For morphology analyses, we took 0.5 μm z-series stacks to generate image projections and obtained images with FV-1000 confocal microscope. For density analyses, spines were defined as 0.5–6-μm lengths, with or without a head, and measured by counting the number of protrusions in 10-μm length of primary dendrites. Spine density was first averaged per neuron, and means from multiple individual neurons were calculated. Morphological assessments of spine density and shape were conducted blindly.

### Stereological tools used for cell counting and quantification

ImageJ software was used for the analyses within the brain slices such as cell counting, axon extension into contralateral cortex (Fig. 5c), dendritic length, and Sholl test. Axon elongation analyses (Fig. 5a) were performed using Adobe Photoshop software.

### Statistical analysis

Results were expressed as means ± SD. When data were obtained from only two groups, Student’s and Welch’s *t* test were used for comparison. Sholl radii or branch orders were analyzed using two-way repeated measures ANOVA with multiple comparisons made using the Tukey-Kramer least significant difference (LSD) test. For other experiments, the rate of cell scores were initially analyzed using the one-way analysis of variance (ANOVA). Subsequently, Tukey-Kramer least significant difference (LSD) test was applied to absolute values as a post hoc test of multiple comparisons. The level of statistical significance was considered to be *p* < 0.05. Statistical analysis was performed using Statcel3 software (OMS Inc., Tokorozawa, Japan).

## Results

### Roles of Rbfox1-iso2 in neuronal positioning during corticogenesis

**Fig. 1:**
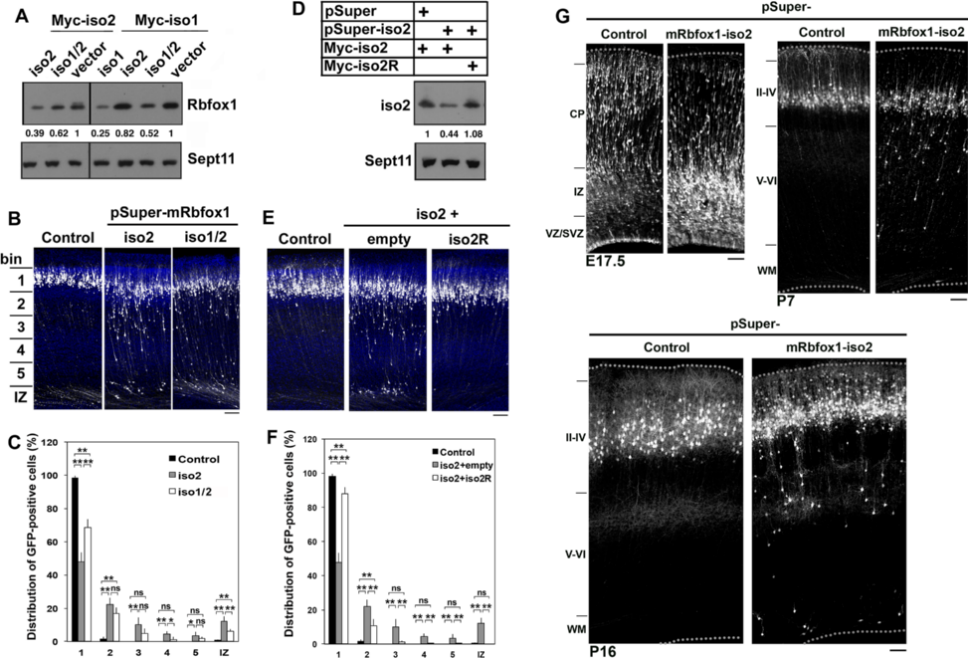
Role of mRbfox1-iso2 in neuronal migration during mouse brain development. **a** Characterization of RNAi vectors. pCS-MT-mRbfox1-iso1 or pCS-MT-mRbfox1-iso2 (Myc-iso1 or Myc-iso2) was transfected into COS7 cells with pSuper control vector (vector), pSuper-mRbfox1-iso1, pSuper-mRbfox1-iso2 or pSuper-mRbfox1-iso1/2. After 48 h, cells were harvested and subjected to western blotting (20 μg protein per lane) with anti-A2BP1(Rbfox1). Anti-Sept11 was used for loading control. The *values* represent relative density of the bands normalized to Sept11 with ImageJ software. The band intensity of the control experiments was defined as 1. **b** Migration defects of mRbfox1-iso2-deficient cortical neurons. pCAG-EGFP was electroporated in utero with pSuper control vector, pSuper-mRbfox1-iso2 (iso2), or pSuper-mRbfox1-iso1/2 (iso1/2) into cerebral cortices at E14.5. Coronal sections were prepared at P3 and immunostained for GFP (*white*) and DAPI (*blue*). *Bar*, 100 μm. **c** Quantification of the distribution of transfected neurons in distinct parts of the cerebral cortex (bin 1–5 and IZ) for each condition shown in **b**. *Error bars* indicate SD; control (*n* = 5), iso2 (*n* = 5), iso1/2 (*n* = 3). **p < 0.01 *p < 0.05 by Tukey-Kramer LSD. **d** Characterization of an RNAi-resistant version, mRbfox1-iso2R. pCAG-Myc-mRbfox1-iso2 (Myc-iso2) or pCAG-Myc-mRbfox1-iso2R (Myc-iso2R) was transfected into COS7 cells with pSuper control vector or pSuper-mRbfox1-iso2. After 48 h, cells were harvested and subjected to western blotting with anti-Myc. The blot was reprobed with anti-Sept11. **e** Rescue experiments of mRbfox1-iso2 knockdown. pCAG-EGFP was coelectroporated in utero with pSuper control vector plus pCAG vector (control) or pSuper-mRbfox1-iso2 together with pCAG vector (empty) or pCAG-Myc-mRbfox1-iso2R (iso2R) into cerebral cortices at E14.5, followed by fixation at P3. Coronal sections were stained as in **b**. *Bar*, 100 μm. **f** Quantification of each condition shown in **e**. Analyses were done as in **c**. *Error bars* indicate SD; control (*n* = 5), iso2 (empty, *n* = 5), iso2 + iso2R (*n* = 5). **p < 0.01 by Tukey-Kramer LSD. **g** Positioning of mRbfox1-iso2-deficient neurons at E17.5, P7, and P16. In utero transfection was done as in **b** at E14.5, followed by fixation at the indicated time points. Coronal sections of control and knockdown samples were immunostained for GFP. *Bars*, 100 μm

We first examined the role of cytoplasmic Rbfox1-iso2 in the migration of newly generated excitatory neurons by acute knockdown in utero. We designed three RNAi vectors, pSuper-mRbfox1-iso1, pSuper-mRbfox1-iso2, and pSuper-mRbfox1-iso1/2, against distinct regions in the *mRbfox1* coding sequences. While Rbfox1-iso2 is highly homologous to Rbfox1-iso, we confirmed that pSuper-mRbfox1-iso2 specifically silenced mRbfox1-iso2 (Fig. 1a). Notably, pSuper-mRbfox1-iso2 had only faint effects on mRbfox1-iso1 expression under the conditions where pSuper-mRbfox1-iso1 silenced the isoform in COS7 cell transfection experiments (Fig. 1a). On the other hand, pSuper-mRbfox1-iso1/2, which targets for a common sequence of Rbfox1-iso1 and Rbfox1-iso2, also knocked down mRbfox1-iso2 under the conditions (Fig. 1a).

pCAG-EGFP was electroporated in utero with pSuper-H1.shLuc control or pSuper-mRbfox1-RNAi vectors into ventricular zone (VZ) progenitor cells of mice brains at E14.5, and localization of the transfected cells and their progeny was visualized at postnatal day (P) 3. While control neurons were positioned normally at the superficial layer (bin 1; layers II~III) of cortical plate (CP), a considerable portion of cells transfected with pSuper-mRbfox1-iso2 or pSuper-mRbfox1-iso1/2 remained in the lower zone of CP and intermediate zone (IZ) (Fig. 1b, c). To examine the specific role of mRbfox1-iso2, we hereafter used pSuper-mRbfox1-iso2 throughout the study. It should be noted that many mRbfox1-iso2-deficient neurons reached the superficial layer (Fig. 1b, c). Transfection efficiency may explain the result; mRbfox1-iso2 was incompletely knocked down in neurons incorporating low amount of the RNAi vector. Rescue experiments were then performed to rule out off-target effects by the use of mRbfox1-iso2R that was resistant to pSuper-mRbfox1-iso2 (Fig. 1d). When pSuper-mRbfox1-iso2 was coelectroporated with pCAG-Myc-mRbfox1-iso2R, the positional defects were rescued at P3 (Fig. 1e, f), indicating that the abnormal positioning observed was indeed caused by reduced expression of mRbfox1-iso2. When we analyzed the effects of mRbfox1-iso2-knockdown on the neuronal migration at E17, positioning defects were detected at this stage; while most control neurons were migrating in CP, many mRbfox1-iso2-deficient neurons were still in IZ (Fig. 1g). We then examined the long-term effects at P7 and P16 and found that many mRbfox1-iso2-deficient cells did not make it to the correct target destination (layers II–IV) at these time points. These results suggest that efficient mRbfox1-iso2-silencing caused defects in the radial migration of cortical neurons.

Since cell morphology is closely associated with migration, we looked into the shape of mRbfox1-iso2-deficient neurons with abnormal positioning at E17.5 (Fig. 1g). Consequently, the deficient neurons transformed smoothly into bipolar status in the upper IZ and had apparently normal bipolar morphology in CP (Fig. 1g; E17.5), suggesting that mRbfox1-iso2 is not directly involved in neuronal morphology before and during migration. However, it should be noted that we here analyzed fixed cells and the obtained results are considered as snapshots at the time point.

### Time-lapse imaging of migration of mRbfox1-iso2-deficient neurons in cortical slices

**Fig. 2:**
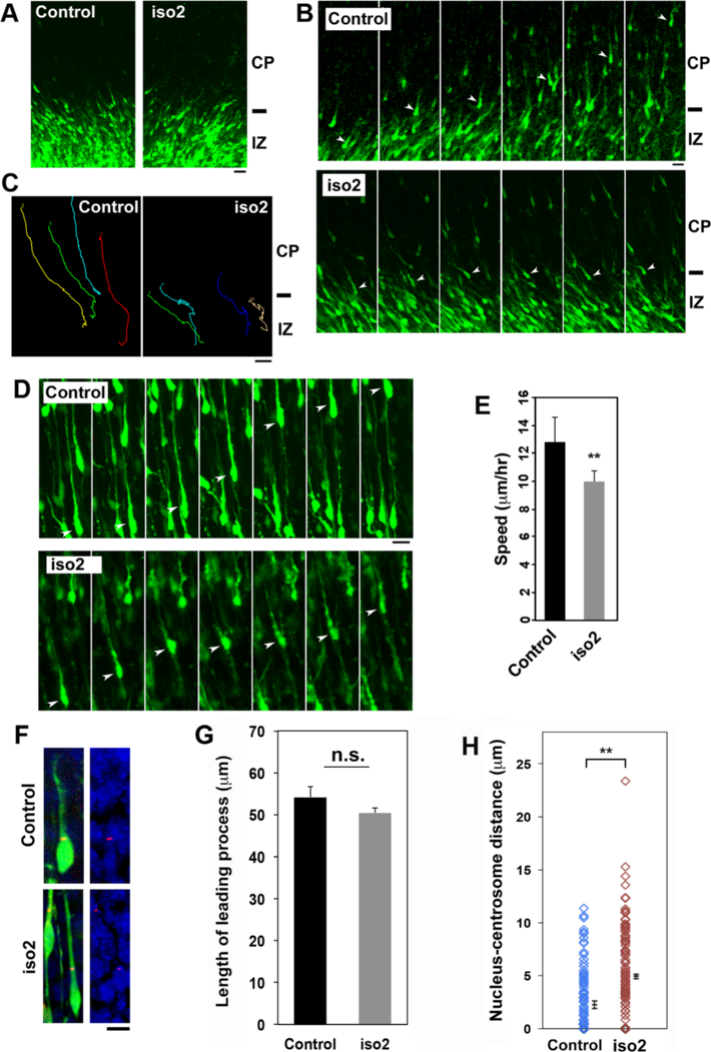
Time-lapse imaging of migration of mRbfox1-iso2-deficient neurons. Analyses were repeated three times for each case, and the migration pattern was observed for 10 cells in each imaging. Representative results were shown in **a**–**d** and **f. a** Confocal images of cortical slices at the beginning of time-lapse imaging. E14.5 cortices were coelectroporated in utero with pCAG-EGFP together with pSuper control vector or pSuper-mRbfox1-iso2 (iso2), followed by coronal section slice preparation at E16.5 and time-lapse imaging (Additional file 1: Video 1 and Additional file 2: Video 2 for control and iso2, respectively). Note that there were no differences in transfection efficiency between the experiments. *Bars* in **a**–**d**, 20 μm. **b** Time-lapse imaging of control and mRbfox1-iso2-deficient neurons (iso2) at the IZ-CP boundary. Magnified images were depicted from Additional file 1: Video 1 (control) and 2 (iso2). **c** Tracing of control or the deficient neurons (iso2) in **b**. Migratory tracks of four representative cells were traced and shown as *color lines*. **d** Time-lapse imaging of control and the deficient neurons migrating in CP (Additional file 3: Video 3 and Additional file 4: Video 4 for control and iso2, respectively). **e** Calculation of migration velocity of control and the deficient neurons in middle-upper CP. Ten cells were analyzed in each experiment (*n* = 3). *Error bars* indicate SD; **p < 0.01 by Student’s *t* test. **f** pCAG-EGFP was electroporated with pCAG-PACKmKO1 together with pSuper control vector or pSuper-mRbfox1-iso2 (iso2) into cerebral cortices at E14.5. Coronal sections were prepared at E17.5 and immunostained with anti-GFP. Centrosome (*red*) and nuclei (*blue*) were also visualized. Representative images of migrating neurons in the lower CP were shown. *Bar*, 5 μm. **g** Quantification of the length of leading process of control and the deficient neurons (iso2) in **f**. Numbers of cells used for calculation in **g** and **h** are 100 in each brain (*n* = 3). *Error bars* indicate SD. **h** Distance between centrosome and the top of nucleus was measured for control and the deficient (iso2) cells. Electroporation was done as in **f**. *Error bars* indicate SD; **p < 0.01 by Student’s *t* test

Since we might miss the important morphological changes in the snapshot data, we next examined the morphology of migrating mRbfox1-iso2-deficient neurons by the use of time-lapse imaging. VZ progenitor cells were coelectroporated with pCAG-EGFP together with the control vector or pSuper-mRbfox1-iso2 at E14.5. When time-lapse imaging was started at E16.5, mRbfox1-iso2-deficient cells were multipolar and some cells were transforming into bipolar neurons as in the case of control cells (Fig. 2a). However, during the observation, migration profile of the deficient cell became abnormal. Control neurons smoothly moved into CP and migrated toward the pial surface after multipolar-bipolar transition in the upper IZ (Fig. 2b, c, *control* panels, and Additional file 1: Video 1). On the other hand, although the deficient cells showed normal multipolar-bipolar transition, they then frequently remained stranded in the upper IZ and subsequent migration was significantly prevented during the imaging time period (~24 h) (Fig. 2b, c, *iso2* panels, and Additional file 2: Video 2).

It is notable that many mRbfox1-iso2-deficient cells still crossed IZ and moved into CP. We thus monitored the migration of such cells in CP. While control cells showed smooth locomotion in CP (Fig. 2d, *control* panels, Additional file 3: Video 3), the deficient cells exhibited characteristic migration defects (Fig. 2d, *iso2* panels, e and Additional file 4: Video 4). While the deficient cells maintained apparently normal bipolar shape during radial migration (Fig. 2f, g), the distance between the nucleus and the preceding centrosome (N-C distance) was abnormally longer, suggestive of the impaired nucleokinesis (Fig. 2h). Nucleokinesis is the process of translocation of the nucleus into the proximal leading process during radial migration and dependent on the microtubule dynamics [33]. Although dynamic changes of the microtubule function significantly affect the radial migration profile and we showed the N-C distance abnormality (Fig. 2h), cell shapes in snapshot data do not satisfactorily reveal changes in the microtubule dynamics and thus time-lapse imaging analyses are very useful. Collectively, mRbfox1-iso2 may be involved in the crossing over the IZ-CP border after multipolar-bipolar transition in the upper IZ and subsequent radial migration in CP. We suppose that the migration defects observed depend on the degree of knockdown; while migration defects might take place at the IZ-CP border when the RNAi effect is strong, the aberrant migration phenotype might be observed in CP after crossing the border when the RNAi effect is relatively weak.

### Involvement of mRbfox1-iso2 in the terminal translocation of cortical neurons

**Fig. 3:**
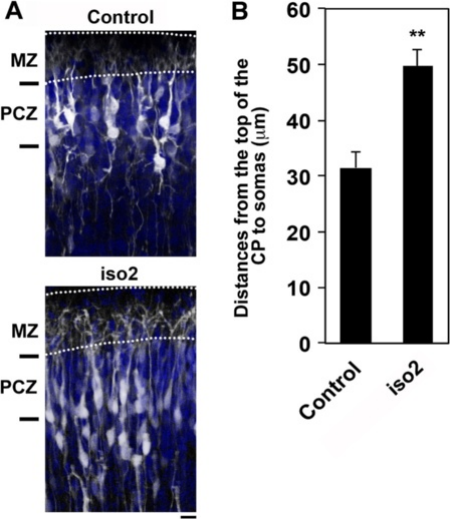
Role of mRbfox1-iso2 in the terminal translocation of cortical neurons. **a** mRbfox1-iso2-knockdown hampers the terminal translocation. Cerebral cortices were electroporated with pCAG-EGFP together with pSuper vector (control) or pSuper-mRbfox1-iso2 (iso2) at E15.5. Coronal sections were prepared at P3 and immunostained with anti-GFP (*white*) and DAPI (*blue*). *Dotted lines* represent the pial surface (*upper*) and top of CP (*lower*). *MZ* marginal zone, *PCZ* primitive cortical zone. **b** Statistical analyses of **a**. Distance between the top of CP and the cell soma was measured. Representative slices form 5 (control) or 4 (iso2) brains were analyzed for each analysis. Numbers of cells used for each calculation were 100. *Error bars* indicate SD; **p < 0.01 by Student’s *t* test

At the end of radial migration in CP, the migratory mode of cortical neurons changes to the terminal translocation, a crucial step for the completion of neuronal migration, just beneath the marginal zone (MZ) [34]. We asked if mRbfox1-iso2-knockdown has some effects on the terminal translocation. As shown in Fig. 3, terminal translocation was not completed when mRbfox1-iso2 was silenced. The deficient neurons could not enter the outermost region of the CP termed primitive cortical zone, although the tip of the leading process reached MZ. These results indicate that mRbfox1-iso2 is involved in the terminal translocation as well as radial migration. Since neuronal somas move quickly in a radial glia-independent manner during the terminal translocation, nucleokinesis is most likely to be essential for the terminal translocation. Therefore, migration defects observed in the radial migration and terminal translocation may be caused by the same molecular mechanism (impaired nucleokinesis), although further molecular analyses are crucial to address this issue.

### Role of mRbfox1-iso2 in the proliferation of neuronal progenitor and stem cells

**Fig. 4:**
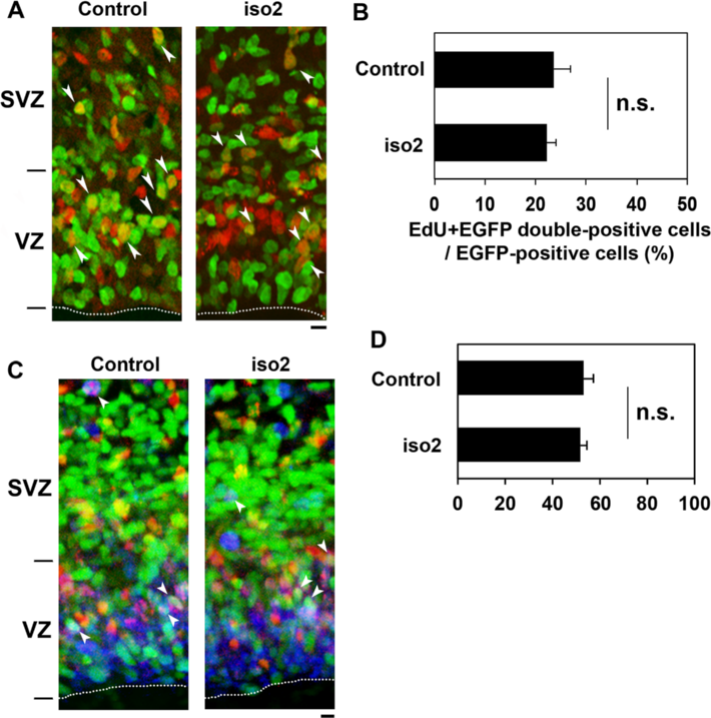
Effects of mRbfox1-iso2-silencing on the cell cycle of VZ/SVZ progenitor and stem cells. **a** Effects of mRbfox1-iso2-silencing on EdU incorporation were examined. Cortices were electroporated in utero with pCAG-H2B-EGFP together with pSuper vector (control) or pSuper-mRbfox1-iso2 (iso2). Coronal sections were visualized for GFP (*green*) and EdU (*red*). *Arrowheads* indicate EdU/GFP double positive cells. *Dotted lines* represent ventricular surface. *Bar*, 5 μm. **b** Quantification of EdU/GFP double positive cells among GFP-positive ones (*n* = 100) in **a**. Two sections were used per brain (control, *n* = 5; iso2, *n* = 4). *Error bars* indicate SD. **c** Effects of mRbfox1-iso2-silencing on cell cycle exit. Differentiated neurons are EdU/GFP double positive while EdU/Ki67/GFP-triple-positive cells maintain progenitor potency. *Arrowheads* indicate triple-positive cells. **d** Quantification of EdU/Ki67/GFP-triple-positive cells among GFP/EdU double positive ones (*n* = 100) in **c**. The ratio of the triple-positive cells over the total double positive ones in mRbfox1-iso2-silencing experiments was similar to that in the control ones. Two sections were used per brain. *Error bars* indicate SD (control, *n* = 3; iso2, *n* = 3)

Since prolonged cell cycle causes delayed cortical neuron migration during corticogenesis [35], we analyzed the effects of mRbfox1-iso2-silencing on the cell cycle of progenitor and stem cells in VZ/subventricular zone (SVZ). When S-phase cells were labeled with EdU to detect DNA replication, mRbfox1-iso2-deficient cells entered S-phase to an extent similar to the control cells (Fig. 4a, b). These results strongly suggest that mRbfox1-iso2 does not participate in the proliferation of VZ/SVZ cells. Also, distribution of the EdU/EGFP-double-positive cells in VZ/SVZ was not altered by the knockdown (Fig. 4a).

We further looked into the effects of mRbfox1-iso2-silencing on the proliferation of neuronal progenitor and stem cells by triple staining for EdU, GFP, and Ki67, a marker for all active phases of the cell cycle except the quiescent G0 state. In this analysis, cells still in proliferating after EdU incorporation could be identified as EdU/Ki67-double-positive while neurons that differentiated after EdU incorporation would be EdU-positive but Ki67-negative. Consequently, between control and mRbfox1-iso2-deficient cells, no statistical differences were observed in the ratio of EdU/Ki67/GFP-triple-positive cells to EdU/GFP-double-positive cells, indicating that mRbfox1-iso2-deficient progenitor and stem cells differentiated to neuronal cells at a rate similar to the control cells (Fig. 4c, d).

Taken together with the result that mRbfox1 was limitedly expressed in CP and upper IZ but not VZ/SVZ during corticogenesis [20], we concluded that the positioning defects by mRbfox1-iso2-silencing resulted from abnormal neuron migration.

### mRbfox1-iso2 regulates axon elongation and dendrite development in vivo

**Fig. 5:**
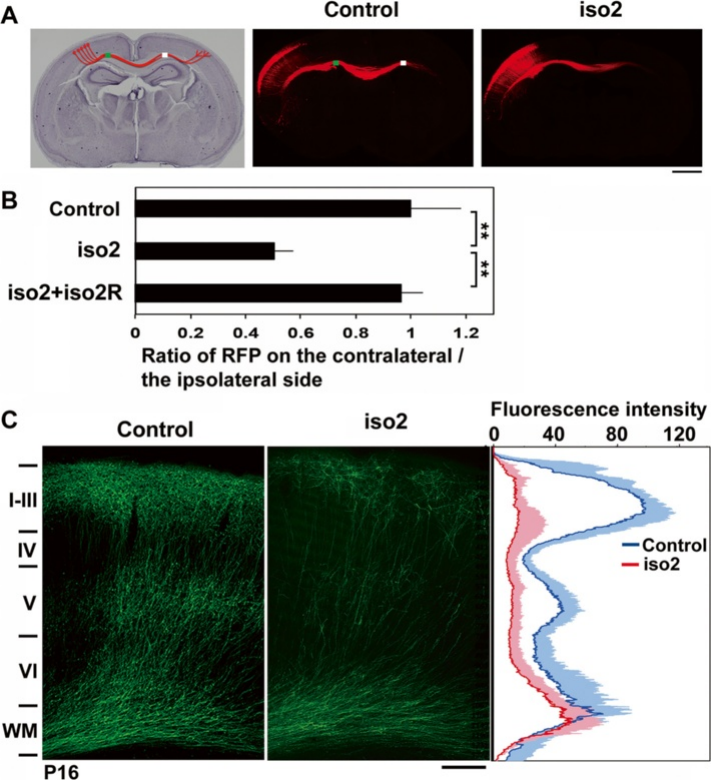
Role of mRbfox1-iso2 in the axon growth of cortical neurons in vivo. **a** pCAG-RFP was electroporated with control pSuper vector (control) or pSuper-mRbfox1-iso2 (iso2) into cerebral cortices at E14.5. Coronal sections were prepared at P3. Hematoxylin staining of a slice was also shown. *Bar*, 1 mm. **b** Quantitative analyses of the ratio of the intensity of RFP-positive axons in the area (*white*) of contralateral cortex to that in the area (*green*) of ipsilateral one in **a**. Rescue experiments were also done by cotransfection with pCAG-Myc-mRbfox1-iso2R (iso2R). *Error bars* indicate SD; control (*n* = 5), iso2 (*n* = 5), iso2 + iso2R (*n* = 4); **p < 0.01 by Tukey-Kramer LSD. **c** Representative images of the terminal arbors of axons expressing GFP with pSuper vector (control) or pSuper-mRbfox1-iso2 at P16. Note that axons of the deficient neurons reached contralateral hemisphere at the time point, whereas they did not extend efficiently into the cortical layer structure. Densitometric analyses of GFP fluorescence intensity were also carried out. *Blue* (control) and *red* (iso2) *lines*, average; *shadow*, SD (control, *n* = 6; iso2, *n* = 6). *Bar*, 200 μm

Synaptic dysfunction and disrupted synaptic network are known to be involved in pathogenesis of neurodevelopmental and psychiatric disorders. We thus looked into the effects of mRbfox1-iso2-silencing on axon elongation into the contralateral hemisphere. Consequently, axon density became lower after leaving the corpus callosum (Fig. 5a, b), and the phenotype was rescued by coexpression of mRbfox1-iso2R (Fig. 5b). Notably, axons from the hemisphere containing the deficient cells eventually ended up elongating and reaching the contralateral white matter at P7 (data not shown) and P16 (Fig. 5c). However, it should be noted that such axons did not extend efficiently into the cortical layer structure even at P16 (Fig. 5c). These results suggest that mRbfox1-iso2 plays a role in the axon growth and extension into the contralateral cortex.

**Fig 6:**
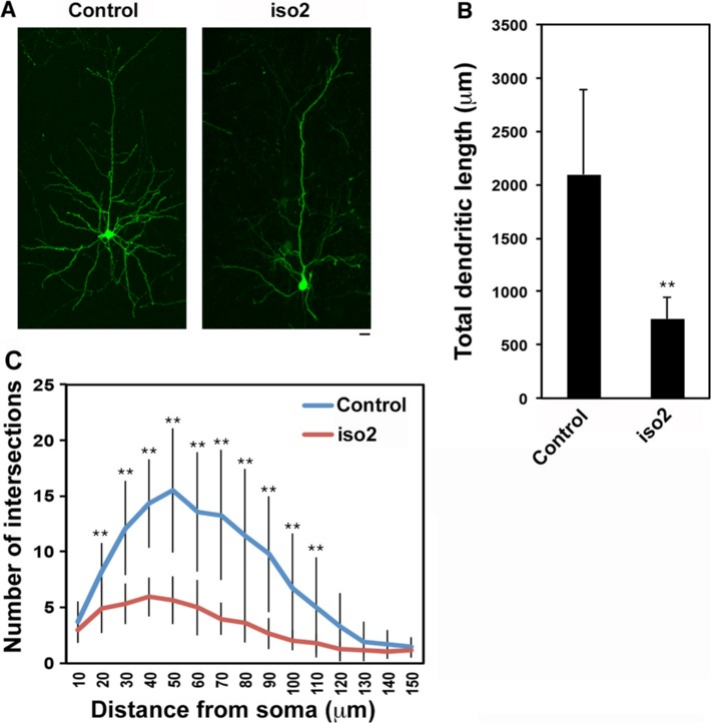
Role of mRbfox1-iso2 in the dendrite growth in cortical neurons in vivo. **a** pCAG-loxP-GFP was electroporated for sparse expression with pCAG-M-Cre together with pSuper vector (Control) or pSuper-mRbfox1-iso2 (iso2) at E14.5. Analyses were carried out in cortical slices at P30. Representative average Z-stack projection images of GFP fluorescence of upper layer cortical neurons were shown. **b**, **c** One section from each brain was analyzed for control (*n* = 4) and iso2 (*n* = 6) experiments. Representative data were shown. **b** Total dendritic length was calculated for neurons observed at P30. *Error bars* indicate SD; **p < 0.01 by Student’s *t* test. **c** Branch points of dendrites were analyzed by Sholl test. *Error bars* indicate SD (*n* = 19 neurons for control, *n* = 20 for iso2); **p < 0.01 by Tukey-Kramer LSD.

We next examined the role of mRbfox1-iso2 in dendritic arbor formation in vivo. Introduction of pSuper-mRbfox1-iso2 at E14.5 into VZ cells resulted in highly abrogated dendritic arborization at P30 (Fig. 6a). As shown in Fig. 6b, c, the total length of dendrites per cell and branching point number at each successive 10-μm Sholl radius were both significantly decreased in mRbfox1-iso2-deficient neurons, strongly suggesting that mRbfox1-iso2 is essential for dendritic arbor formation and maintenance. Taken together, functional loss of mRbfox1-iso2 is most likely to impair neuronal connectivity through defective axon and dendrite development.

### Involvement of mRbfox1-iso2 in spine morphology in vitro

**Fig. 7:**
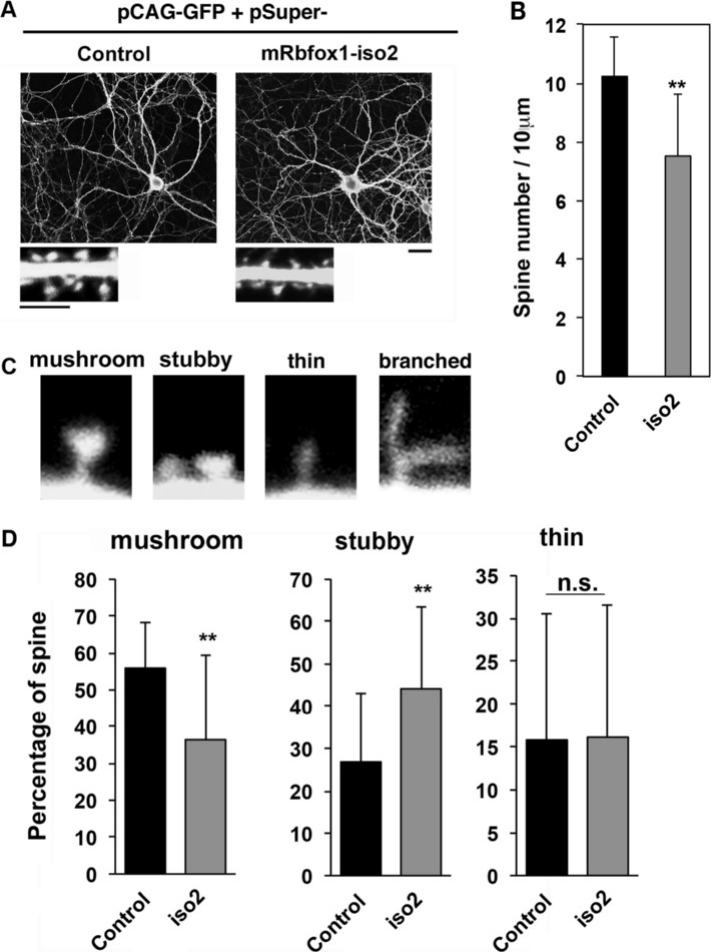
Role of mRbfox1-iso2 in the dendritic spine morphology of primary cultured hippocampal neurons. **a** Neurons were transfected with pβAct-EGFP together with pSuper vector (control) or pSuper-mRbfox1-iso2 (iso2) when isolated, fixed at 21 div and stained for GFP. *Bar*, 20 μm. Magnified images of dendrites are shown. *Bars*, 10 μm (*upper panels*) and 5 μm (*lower panels*). **b** Quantitative analyses of density of dendritic spines for each condition in **a**. *Error bars* show SD of the results from control (*n* = 17) and iso2-transfected neurons (*n* = 23). Experiments were repeated three times with similar results and representative data were shown. **p < 0.01 by Student’s *t* test. **c** Typical examples of mature (mushroom) and immature (stubby, thin filopodia, and branch) spines. **d** Relative abundance of the four different spine types in neurons transfected with pSuper vector (control) or pSuper-mRbfox1-iso2 (iso2) was analyzed. Relative percentages of spine types were indicated in graphs. *Error bars* show SD of the results from 150 spines (control, 17 cells; iso2, 23 cells). Experiments were repeated three times with similar results and representative data were shown. **p < 0.01 by Student’s *t* test. Note that branched spine was hardly detected under the conditions used (less than 2 % of total spine number)

To further test the possibility of the involvement of mRbfox1-iso2 in the spine morphology, we carried out in vitro experiments. To this end, primary cultured mouse hippocampal neurons were transfected with pβAct-EGFP together with the control vector or pSuper-mRbfox1-iso2 immediately after isolation, fixed at 21 div (days in vitro) and stained for GFP. Under the conditions, spine density was decreased in neurons transfected with pSuper-mRbfox1-iso2 (Fig. 7a, b). We then looked into the spine morphogenesis in the deficient neurons by counting four established spine morphology groups (i.e., mushroom, stubby, thin filopodia-like, and branched spines) (Fig. 7c). When compared to the control neurons, relative percentage of mushroom (mature) spine decreased and that of stubby spine increased concomitantly in the deficient neurons (Fig. 7d). It is notable that the ratio of the branched spine was less than 2 % in these assay conditions.

## Discussion

ASD is highly heritable, and the genetic basis has been pursued intensively using genetic and genomic analyses, which have identified a variety of genetic causes that account for ~20 % of ASD cases, including genetic copy number variation (CNV), syndromic forms of ASD, and single-gene and metabolic disorders [36].

*RBFOX1* has been shown to play a central “hub” role in the ASD gene transcriptome network [17]. mRbfox1 functions as an RNA splicing factor and is expressed in differentiated and migrating neurons in developing mouse cerebral cortex and is excluded from cells in proliferative zones based on both in situ hybridization (E15) [37] and immunohistochemistry (E17) [20]. Since cell biological relevance of RBFOX1 in the brain development has been enigmatic, we carried out comprehensive in vivo and in vitro analyses to obtain information to understand the etiology of ASD and other neurodevelopmental disorders in which *RBFOX1* gene abnormalities are involved. While there are seven *RBFOX1* mRNA variants encoding total six isoforms (variants 4 and 6 both encode isoform 4) based on the NCBI database (http://www.ncbi.nlm.nih.gov/gene/54715), we here focused on the brain-specific cytosolic isoform, Rbfox1-iso2. Inclusion and exclusion of exon A53 result in Rbfox1-iso1 and Rbfox1-iso2, respectively, with different amino acid sequences at the C-terminal regions owning to a frame shift [19]. While Rbfox1-iso1 is the major Rbfox1 isoform residing in the nucleus, Rbfox1-iso2 is a relatively minor isoform which distributes in the cytoplasm [19, 20]. It is most likely that the specific distribution of Rbfox1-iso1 and Rbfox1-iso2 in the nucleus and cytosol, respectively, should be attributable to the C-terminal sequence difference.

When mRbfox1-iso2 was silenced in utero and the phenotypes were examined, defects in radial migration, terminal translocation, axon elongation to the contralateral cortex, and dendritic arbor formation were observed. These results imply functional impairment of the cytoskeleton system in the deficient neurons. In this context, *FLNA*-encoding actin-binding protein FilaminA and *RHOA*-encoding small GTPase RhoA have been reported to be downstream splicing candidates for RBFOX1 [38]. In addition, RBFOX1 is possible to regulate expression of *DCX* encoding microtubule-binding protein and *CDK5R1* encoding regulatory subunit of CDK5 kinase [38]. On the other hand, cell cycle (proliferation) of VZ/SVZ cells was not affected by mRbfox1-knockdown, consistent with the observation that mRbfox1 is predominantly expressed in neurons but not in progenitor and stem cells in the proliferative zone.

It is also notable that in vitro experiments strongly suggest that Rbfox1-iso2 serves as a regulator for spine morphology and dynamics; immature (stubby) spine was increased when mRbfox1-iso2 was silenced in primary cultured hippocampal neurons. Since neurodevelopmental disorders such as ASD and schizophrenia are thought to be “synapse” diseases, these results suggest a crucial role of Rbfox1-iso2 in not only the developing but also the adult brain. Together with the report that mRbfox1-expressing neurons were distributed throughout the cortex when the cortical formation completed [20], *RBFOX1* gene abnormalities may influence the clinical features of ASD and other neurodevelopmental disorders in the adult stage through disruption of synapse functions [6–8].

*RBFOX1*-downregulation has been shown in a subset of ASD patient brains, leading to splicing changes of genes encoding various proteins essential for cytoskeleton, scaffolding, synaptic transmission, ion channels, and transcription regulation [18]. Meanwhile, further intensive analyses are required to address this issue, since no significant difference in *RBFOX1* expression in lymphoblastoid cell lines has been reported between ASD cases and controls sibs [39]. While *RBFOX1* gene abnormalities may form common pathophysiological background in the patients, aberrant splicing is possible to occur in a manner which depends on the genetic background of each patient and might be related to the pathophysiology of divergent clinical symptoms in ASD. In either case, it seems to be crucial to find not only yet unidentified splicing targets but also environmental and/or genetic modification factors which may explain the complexity of etiology and clinical symptoms of ASD involving *RBFOX1* gene abnormalities.

Considering that splicing and transcription occur in the nucleus, Rbfox1-iso2 is supposed to exert its function through shuttling between the nucleus and cytoplasm as in the case of a group of splicing factors called heterogeneous nuclear ribonucleoproteins (hnRNPs) [40]. Interestingly, hnRNPs are known to have additional cytoplasmic roles in mRNA-transport, mRNA-localization, mRNA-turnover, and regulation of translation [40, 41]. It is thus tempting to speculate that Rbfox1-iso2 participates in cytoplasmic events as well as RNA splicing in the nucleus. It should be noted that similar but not identical phenotypes were obtained in the neuronal cell migration and morphology when mRbfox1-iso1 was knocked down.^1^ The phenotype difference in the two isoforms might be explained by their distinct functions, such as yet unidentified cytoplasmic role of Rbfox1-iso2, based on the different localization and/or C-terminal amino acid structure. On the other hand, transient nuclear distribution of Rbfox1-iso2 by shuttling might be important for its RNA splicing function and explain the phenotype similarity to Rbfox1-iso1.

From the observations in this study, mRbfox1-iso2 was shown to be involved in the regulation of various processes essential for the cortical development. A variety of aberrant phenotypes observed during corticogenesis (defects in migration, axon growth, and dendrite arborization) and in vitro analyses (defects in spine morphology) may be the consequence of the impaired splicing events in affected neurons and could account for the emergence of the various clinical symptoms of ASD and other disorders such as ADHD, ID, epilepsy, and schizophrenia, where *RBFOX1* gene abnormalities are involved. Molecular aspects of impaired splicing events in Rbfox1-deficient neurons remain to be clarified. Aberrant phenotypes observed in this study are also supposed to be involved in the various clinical symptoms of ASD and other neurodevelopmental disorders. Further intensive studies to elucidate the molecular bases of physiological and pathophysiological roles of mRbfox1 isoforms are essential to understand the commonality and diversity of the abovementioned disorders.

## Conclusions

Gene abnormalities in *RBFOX1*, encoding an mRNA-splicing factor, have been shown to cause ASD and other neurodevelopmental and psychiatric disorders such as ID, ADHD, and schizophrenia. Notably, *RBFOX1* was recently found to serve as a “hub” in ASD gene transcriptome networks by a sophisticated system biology approach, strongly suggesting the key role of this gene in the pathophysiology common to the above disorders. Since the physiological role of RBFOX1 in cortical development is still largely unknown, it is essential to elucidate the function in corticogenesis for understanding the pathophysiological significance of the molecule in ASD and related disorders.

To elucidate the pathophysiological relevance of RBFOX1, we focused on the cytoplasmic isoform of Rbfox1 (Rbfox1-iso2) and examined its role during mouse corticogenesis in vivo and in vitro. Knockdown of Rbfox1-iso2 in vivo caused defects in the initiation and maintenance of radial migration and the completion of terminal translocation of cortical neurons. Rbfox1-iso2 was also found to regulate synapse network formation since axon growth and dendritic arborization were abrogated when this molecule was silenced in vivo. In vitro experiments revealed that spine density and mature spine number were reduced in Rbfox1-iso2-deficient primary cultured hippocampal neurons. Abnormal phenotypes observed were similar but not the same as those in the nuclear isoform Rbfox1-iso1-deficient neurons.

Consequently, we found an essential role of a cytosolic isoform of Rbfox1, Rbfox1-iso2, in the cortical development. Functional abnormalities in Rbfox1-iso2-deficient neurons may induce structural and functional defects of the cerebral cortex and consequently contribute to the clinical symptoms of ASD and other neurodevelopmental and psychiatric disorders with *RBFOX1* gene abnormalities.

## Additional files

Additional file 1: Video 1. Time-lapse imaging of control neurons migrating in upper IZ to lower CP. Neurons smoothly moved into CP and migrated toward the pial surface after multipolar-bipolar transition in the upper IZ. Additional file 2: Video 2. Time-lapse imaging of Rbfox1-iso2-defcient neurons stranded in upper IZ to lower CP. The deficient cells showed normal multipolar-bipolar transition. Howevwe, they then frequently remained stranded in the upper IZ and subsequent migration was significantly prevented during the imaging time period. Additional file 3: Video 3. Time-lapse imaging of control neurons migrating in CP. Control cells showed smooth locomotion in CP. Additional file 4: Video 4. Time-lapse imaging of Rbfox1-iso2-defcient neurons migrating in CP. The deficient cells exhibited characteristic migration defects.

## Abbreviations

ADHD: Attention deficit hyperactivity disorder
ASD: Autism spectrum disorder
CP: Cortical plate
EdU: 5-ethynyl-2′-deoxyuridine
ID: Intellectual disability
IZ: Intermediate zone
MZ: Marginal zone
PCZ: Primitive cortical zone
SVZ: Subventricular zone
VZ: Ventricular zone

## Footnotes

1 The experimental results will be described elsewhere.

## References

[1] Shibata H, Huynh DP, Pulst SM (2000). A novel protein with RNA-binding motifs interacts with ataxin-2. Hum Mol Gen.

[2] Lee JA, Tang ZZ, Black DL (2009). An inducible change in Fox-1/A2BP1 splicing modulates the alternative splicing of downstream neuronal target exons. Genes Dev.

[3] Gehman LT, Stoilov P, Maguire J, Damianov A, Lin C-H, Shiue L, Ares M, Mody I, Black DL (2011). The splicing regulator Rbfox1 (A2BP1) controls neuronal excitation in the mammalian brain. Nat Genet.

[4] Zhou HL, Baraniak AP, Lou H (2007). Role for Fox-1/Fox-2 in mediating the neuronal pathway of calcitonin/calcitonin gene-related peptide alternative RNA processing. Mol Cell Biol.

[5] Tang ZZ, Zheng S, Nikolic J, Black DL (2009). Developmental control of CaV1.2 L-type calcium channel splicing by fox proteins. Mol Cell Biol.

[6] Bhalla K, Phillips HA, Crawford J, McKenzie OLD, Mulley JC, Eyre H, Gardner AE, Kremmidiotis G, Callen DF (2004). The de novo chromosome 16 translocations of two patients with abnormal phenotypes (mental retardation and epilepsy) disrupt the A2BP1 gene. J Hum Genet.

[7] Elia J, Gai X, Xie HM, Perin JC, Geiger E, Glessner JT, D’arcy M, de Berardinis R, Frackelton E, Kim C, Lantieri F, Muganga BM, Wang L, Takeda T, Rappaport EF, Grant SF, Berrettini W, Devoto M, Shaikh TH, Hakonarson H, White PS (2009). Rare structural variants found in attention-deficit hyperactivity disorder are preferentially associated with neurodevelopmental genes. Mol Psychiatry.

[8] Xu B, Roos JL, Levy S, van Rensburg EJ, Gogos JA, Karayiorgou M (2008). Strong association of de novo copy number mutations with sporadic schizophrenia. Nat Genet.

[9] Hamshere ML, Green EK, Jones IR, Jones L, Moskvina V, Kirov G, Grozeva D, Nikolov I, Vukcevic D, Caesar S, Gordon-Smith K, Fraser C, Russell E, Breen G, St Clair D, Collier DA, Young AH, Ferrier IN, Farmer A, McGuffin P, Hamshere ML, Green EK, Jones IR, Jones L, Moskvina V, Kirov G, Grozeva D, Nikolov I, Vukcevic D, Caesar S, Gordon-Smith K, Fraser C, Russell E, Breen G, St Clair D, Collier DA, Young AH, Ferrier IN, Farmer A, McGuffin P, Holmans PA, Owen MJ, O’Donovan MC, Craddock N, Wellcome Trust Case Control Consortium (2009). Genetic utility of broadly defined bipolar schizoaffective disorder as a diagnostic concept. Br J Psychiatry.

[10] Bucan M, Abrahams BS, Wang K, Glessner JT. Genome-wide analyses of exonic copy number variants in a family-based study point to novel autism susceptibility genes. PLoS Genet. 2009. 10.1371/journal.pgen.1000536 PMC2695001 19557195

[11] Gai X, Xie HM, Perin JC, Takahashi N, Murphy K, Wenocur AS, D’arcy M, O’Hara RJ, Goldmuntz E, Grice DE, Shaikh TH, Hakonarson H, Buxbaum JD, Elia J, White PS (2012). Rare structural variation of synapse and neurotransmission genes in autism. Mol Psychiatry.

[12] Pinto D, Pagnamenta AT, Klei L, Anney R, Merico D, Regan R, Conroy J, Magalhaes TR, Correia C, Abrahams BS, Almeida J, Bacchelli E, Bader GD, Bailey AJ, Baird G, Battaglia A, Berney T, Bolshakova N, Bölte S, Bolton PF, Bourgeron T, Brennan S, Brian J, Bryson SE, Carson AR, Casallo G, Casey J, Chung BHY, Cochrane L, Corsello C (2010). Functional impact of global rare copy number variation in autism spectrum disorders. Nature.

[13] Sanders SJ, Ercan-Sencicek AG, Hus V, Luo R, Murtha MT, Moreno-De-Luca D, Chu SH, Moreau MP, Gupta AR, Thomson SA, Mason CE, Bilguvar K, Celestino-Soper PBS, Choi M, Crawford EL, Davis L, Wright NRD, Dhodapkar RM, DiCola M, DiLullo NM, Fernandez TV, Fielding-Singh V, Fishman DO, Frahm S, Garagaloyan R, Goh GS, Kammela S, Klei L, Lowe JK, Lund SC (2011). Multiple recurrent de novo CNVs, including duplications of the 7q11.23 Williams syndrome region, are strongly associated with autism. Neuron.

[14] Buxbaum JD, Silverman J, Keddache M, Smith CJ, Hollander E, Ramoz N, Reichert JG (2004). Linkage analysis for autism in a subset families with obsessive-compulsive behaviors: evidence for an autism susceptibility gene on chromosome 1 and further support for susceptibility genes on chromosome 6 and 19. Mol Psychiatry.

[15] International Molecular Genetic Study of Autism Consortium (IMGSAC) (2001). A genomewide screen for autism: strong evidence for linkage to chromosomes 2q, 7q, and 16p. Am J Hum Genet.

[16] Zhang C, Frias MA, Mele A, Ruggiu M, Eom T, Marney CB, Wang H, Licatalosi DD, Fak JJ, Darnell RB (2010). Integrative modeling defines the nova splicing-regulatory network and its combinatorial controls. Science.

[17] Voineagu I, Wang X, Johnston P, Lowe JK, Tian Y, Horvath S, Mill J, Cantor RM, Blencowe BJ, Geschwind DH (2011). Transcriptomic analysis of autistic brain reveals convergent molecular pathology. Nature.

[18] Weyn-Vanhentenryck SM, Mele A, Yan Q, Sun S, Farny N, Zhang Z, Xue C, Herre M, Silver PA, Zhang MQ, Krainer AR, Darnell RB, Zhang C (2014). HITS-CLIP and integrative modeling define the Rbfox splicing-regulatory network linked to brain development and autism. Cell Reports.

[19] Nakahata S, Kawamoto S (2005). Tissue-dependent isoforms of mammalian Fox-1 homologs are associated with tissue-specific splicing activities. Nucleic Acids Res.

[20] Hamada N, Ito H, Iwamoto I, Mizuno M, Morishita R, Inaguma Y, Kawamoto S, Tabata H, Nagata K-I (2013). Biochemical and morphological characterization of A2BP1 in neuronal tissue. J Neurosci Res.

[21] Kim KK, Kim YC, Adelstein RS, Kawamoto S (2011). Fox-3 and PSF interact to activate neural cell-specific alternative splicing. Nucleic Acids Res.

[22] Konno D, Shioi G, Shitamukai A, Mori A, Kiyonari H, Miyata T, Matsuzaki F (2007). Neuroepithelial progenitors undergo LGN-dependent planar divisions to maintain self-renewability during mammalian neurogenesis. Nat Cell Biol.

[23] Kawabata I, Umeda T, Yamamoto K, Okabe S (2004). Electroporation-mediated gene transfer system applied to cultured CNS neurons. Neuroreport.

[24] Koresawa Y, Miyagawa S, Ikawa M, Matsunami K, Yamada M, Shirakura R, Okabe M (2000). Synthesis of a new Cre recombinase gene based on optimal codon usage for mammalian systems. J Biochem.

[25] Nishitsuji H, Ikeda T, Miyoshi H, Ohashi T, Kannagi M, Masuda T (2004). Expression of small hairpin RNA by lentivirus-based vector confers efficient and stable gene-suppression of HIV-1 on human cells including primary non-dividing cells. Microbes Infect.

[26] Hanai N, Nagata K-I, Kawajiri A, Shiromizu T, Saitoh N, Hasegawa Y, Murakami S, Inagaki M (2004). Biochemical and cell biological characterization of a mammalian septin, Sept11. FEBS Lett.

[27] Nagata K-I, Ito H, Iwamoto I, Morishita R, Asano T (2009). Interaction of a multi-domain adaptor protein, vinexin, with a Rho-effector, Rhotekin. Med Mol Morphol.

[28] Shinoda T, Ito H, Sudo K, Iwamoto I, Morishita R, Nagata K-I (2010). Septin 14 is involved in cortical neuronal migration via interaction with septin 4. Mol Biol Cell.

[29] Ito H, Morishita R, Shinoda T, Iwamoto I, Sudo K, Okamoto K, Nagata K (2010). Dysbindin-1, WAVE2 and Abi-1 form a complex that regulates dendritic spine formation. Mol Psychiatry.

[30] Tabata H, Nakajima K (2001). Efficient in utero gene transfer system to the developing mouse brain using electroporation: visualization of neuronal migration in the developing cortex. Neuroscience.

[31] Inaguma Y, Hamada N, Tabata H, Iwamoto I, Mizuno M, Nishimura YV, Ito H, Morishita R, Suzuki M, Ohno K, Kumagai T, Nagata KI (2014). SIL1, a causative cochaperone gene of Marinesco-Sjogren syndrome, plays an essential role in establishing the architecture of the developing cerebral cortex. EMBO Mol Med.

[32] Uchino S, Hirasawa T, Tabata H, Gonda Y, Waga C, Ondo Y, Nakajima K, Kohsaka S (2010). Inhibition of N-methyl-d-aspartate receptor activity resulted in aberrant neuronal migration caused by delayed morphological development in the mouse neocortex. Neuroscience.

[33] Tsai L-H, Gleeson JG (2005). Nucleokinesis in neuronal migration. Neuron.

[34] Nadarajah B, Brunstrom JE, Grutzendler J, Wong RO, Pearlman AL (2001). Two modes of radial migration in early development of the cerebral cortex. Nat Neurosci.

[35] Friocourt G, Kanatani S, Tabata H, Yozu M, Takahashi T, Antypa M, Raguenes O, Chelly J, Ferec C, Nakajima K, Parnavelas JG (2008). Cell-autonomous roles of ARX in cell proliferation and neuronal migration during corticogenesis. J Neurosci.

[36] Berg JM, Geschwind DH (2012). Autism genetics: searching for specificity and convergence. Genome Biol.

[37] Hammock EAD, Levitt P (2011). Developmental expression mapping of a gene implicated in multiple neurodevelopmental disorders, *A2bp1 (Fox1)*. Dev Neurosci.

[38] Fogel BL, Wexler E, Wahnich A, Friedrich T, Vijayendran C, Gao F, Parikshak N, Konopka G, Geschwind DH (2012). RBFOX1 regulates both splicing and transcriptional networks in human neuronal development. Hum Mol Gen.

[39] Prandini P, Zusi C, Malerba G, Itan, Pignatti PF, Trabetti E (2014). Analysis of RBFOX1 gene expression in lymphoblastoid cell lines of Italian discordant autism spectrum disorders sib-pairs. Mol Cell Probes.

[40] Shyu AB, Wilkinson MF (2000). The double lives of shuttling mRNA binding proteins. Cell.

[41] Dreyfuss G, Kim VN, Kataoka N (2002). Messenger-RNA-binding proteins and the messages they carry. Nat Rev Mol Cell Biol.

